# Vehicle game lane-changing mechanism and strategy evolution based on trajectory data

**DOI:** 10.1038/s41598-025-89567-z

**Published:** 2025-02-09

**Authors:** Dayi Qu, Kedong Wang, Shouchen Dai, Yicheng Chen, Shanning Cui, Yuxiang Yang

**Affiliations:** 1 School of Mechanical and Automotive Engineering, Qingdao University of Technology, Qingdao, 266520 China; 2 School of Intelligent Manufacturing, Qingdao Huanghai University, Qingdao, 266427 China; 3 School of Civil Engineering, Qingdao University of Technology, Qingdao, 266520 China

**Keywords:** Autonomous vehicle, Lane-changing behavior, Game interaction properties, Trajectory data, Mechanical engineering, Statistics

## Abstract

To improve the safety of ramp vehicles changing lane and to shorten the merging distance, this paper explores the dynamic game interaction properties of vehicles merging and the consistency of vehicles’ decision-making behaviors at the macro-microscopic levels. Using the exiD dataset and evolutionary game theory, the merging behavior of ramp vehicles is modeled to explore the effects of different driving states on the evolutionary convergence of strategies. Based on the game cost theory, the lane choice behavior of mainline vehicles is modeled. Validated by SUMO software, the results show that the model in this paper can significantly improve the safety of vehicle merging and reduce the merging distance. The mainline vehicles are more inclined to change lane and cut out in advance when facing the ramp vehicles under the influence of the change of advantage in the subsequent game.

##  Introduction

The vehicle following and lane changing are common micro-driving behaviors in road traffic, reflecting the decision-making behavior process of vehicles under different driving intentions. With the production and promotion of new energy vehicles and the popularization and application of Connected Autonomous Vehicle, autonomous vehicle following functions such as lane keeping and adaptive cruise control are gradually maturing and showing their advantages^1,2^, while vehicle lane changing requires comprehensive traffic environment information and dynamic interaction with surrounding vehicles to make appropriate decision-making behaviors, which has more complex individual interaction characteristics compared with vehicle following behaviors, therefore, autonomous vehicle lane changing has been a hot research topic in the field of autonomous driving. Therefore, the autonomous lane-changing function of vehicles has always been a hot research topic in the field of autonomous driving.

Compared with the pure mathematical modeling approach, based on the vehicle interaction features hidden in the trajectory data, it can provide an effective and real reference value for modeling the interaction behavior of vehicles. Trajectory datasets record the optimal decisions made by drivers in different traffic situations, and these features can reflect the decision-making tendency of drivers, which can help us better study the driving needs of drivers. Since the trajectory dataset is usually recorded using a drone, we can analyze the overall traffic flow from a macro perspective and further analyze the micro behavior of drivers, which will help us to explore the consistency of vehicle decision-making behavior at the macro-microscopic levels.

## Literature review

### Vehicle merging modelling based on upper-level control

Based on the macro perspective and the goal of optimizing the access efficiency of the converging road sections, some scholars optimize the merging sequence of mainline and merging vehicles through scheduling algorithms or collaborative control strategies and allocate appropriate spatial and temporal rights of way for vehicles to alleviate the path conflict problem of vehicles. Hao et al.^3^ proposed a heuristic rule-based vehicle merging sequence scheduling algorithm based on the hierarchical collaboration framework, which makes up for the shortcomings of the fixed merging sequence that cannot adapt to the driver’s vehicle perturbation to the traffic flow. Liu et al.^4^ considered the uneven characteristics of multi-lane traffic flow distribution and mitigated the problem of local density surge or traffic flow saturation by assigning optimal approach lane to mainline vehicles by establishing a distributed lane selection and centralized cooperative control framework for on-ramp vehicle convergence. Ma et al.^5^ constructed the mainline speed control and ramp cooperative control strategy with the overall efficiency and safety of the converging roadway section as the goal, and constructed the motorway traffic state prediction model based on the macroscopic traffic flow theory.

Some scholars optimize the merging behavior of vehicles by targeting the macro-indicators of traffic flow. Zhu et al.^6^ proposed an upper-level control strategy to coordinate the two traffic streams at on-ramp merging through proactive gap creation and platoon formation. The findings suggest that the coordination scheme is suitable for practical application in real-world scenarios. It has the potential to greatly improve the efficiency of on-ramp merging, especially during periods of high traffic volume. This approach helps to prevent recurring traffic congestion and increases the merging throughput. Jing et al.^7^ proposed a hierarchical and decentralized cooperative coordination framework was developed to systematically control the merging of vehicles. For upper-level control, an optimal control-based algorithm considering input constraints was presented to optimize fuel consumption and passenger comfort. The results demonstrate the potential applicability of cooperative control methods based on upper-level vehicle control.

Obviously, the upper layer or collaborative model based on macro modeling perspective relies more on the collaborative control among vehicles and lacks the process of portraying the micro-dynamic interaction behavior of vehicles. Therefore, it is also important to show the characteristics of the micro interaction behavior of vehicles in the modeling process.

### Vehicle merging model based on micro-interaction behavior

Another part of scholars based on the perspective of micro-interaction behavior to reduce the risk of vehicle merging by optimizing the interaction process of vehicle lane changing, and then completing the process of merging. Lyu et al.^8^ introduced a factor graph framework to model the dependencies between observations and to predict the intentions of other vehicles. This approach improves the overall performance of intention estimation, specifically in terms of collision rates and the average distance between vehicles after merging, thereby enhancing both safety and efficiency. Unlike traditional methods that rely on human-designed models or cost functions, real-world trajectories are employed to approximate the model. Qi et al.^9^ proposed an active gap adaptation collaborative lane-changing model for hybrid driving scenarios based on the advantage of interaction and collaboration of Connected Autonomous Vehicle, which achieves the overall constraints of the vehicle group by dividing the collaborative vehicle group, planning the lane changing sequence, and speed control methods, providing lane changing gaps for lane-changing vehicles and improving the road capacity. Wang et al.^10^ established a dynamic collaborative lane-changing model by controlling the acceleration behavior of the vehicle in the target lane to reduce the impact of lane changing on the downstream vehicle. The results show that the model improves the success rate of lane changing and smoothed the trajectory of the downstream vehicle, but the scope of the collaboration is limited to the merging vehicle and the target lane, and the scope of the interaction is restricted, which makes the speed of the front vehicle to be more oscillating.

with the cross-development of socio-economics and game theory with the field of intelligent transportation, expanding the traditional communication field research in the field of vehicle lane changing is generally divided into the free and mandatory lane changing behaviors, wherein the collaborative lane changing is usually based on the overall revenue maximization to make decision guidance for vehicles, while non-collaborative lane changing models make individual decision judgment with the goal of their gain. Similarly, some studies have shown that the game model can better show the dynamic interaction process between vehicles in the micro lane changing model. Qu et al.^11^ established a game model for vehicle decision-making in mixed traffic environments by considering dynamic risks, and the proposed model has higher road utilization and safety. Ma et al.^12^ established a tripartite game model for multi-lane ramp merging area with the control objective of maximizing overall efficiency, and the results showed that the cooperative model can effectively reduce the conflict time and improve the traffic efficiency. Jing et al.^13^ proposed a collaborative optimization framework and algorithm based on a multi-vehicle game, which has great potential to reduce fuel consumption and travel time as well as improve passenger comfort and transportation efficiency. Xiao et al.^14^ proposed a merging decision-making framework, which involves a Mixer based Trajectory Prediction Model (Mixer-TPM) and a Trajectory Prediction-based Game-theory Decision-Making model (TP-GDM). The results indicate that the Mixer-TPM can improves prediction accuracy significantly compared to the state-of-the-arts and has better computational efficiency. Additionally, TP-GDM shows a high level of similarity to human drivers. Yang et al.^15^ introduced a centralized collaborative control strategy for multi-lane systems based on cooperative game theory. By applying the Pontryagin principle, they derived an analytical solution for the longitudinal optimal control of all vehicles. The effectiveness of the proposed approach was validated through simulations conducted under random traffic conditions. The trajectory data hides the potential characteristics of vehicle interaction behavior, and the mining of vehicle behavior tendency can provide real basis and reference for vehicle interaction behavior modeling.

Vehicle lane-changing and interaction behavior based on a microscopic perspective models aim to optimize vehicle decision-making behavior, which in some cases cannot adequately characterize the operation of traffic flow. Although the simulation results of the existing studies, such as vehicle speed and traffic flow, can reflect the advantages of the micro interaction and game behavior models, there is a gap in the research on the consistency of the intrinsic decision-making mechanism of the macro and micro behavior models, and the simulation and evaluation indicators at the macroscopic level cannot reflect the influence of the vehicle by macroscopic factors.

### Classification of vehicle merging types

Vehicle merging is the expansion of lane-changing behavior in certain scenarios, and scholars usually mine the characteristics of lane-changing behavior based on trajectory data and convergence process and portray the vehicle lane-changing behavior through mathematical models. Zhang et al.^16^ classified vehicle interactions in ramp merge zone scenarios into 9 types by extracting vehicle collision time and following time distances, and analyzed the driver risk perception state for different vehicle merging interaction behavior types. The nine types of vehicle interactions included close following, compressed following, direct forward cut-in, direct rear cut-in, collaborative forward cut-in, collaborative rear cut-in, aggressive forward cut-in, aggressive rear cut-in, and lateral approach. The different types of interaction models reflect drivers’ judgments about the trade-off between driving efficiency and safety risks during the lane changing decision-making process. In addition to the above nine common interaction models, Ye et al.^17^ based on trajectory data analysis and mining found that there is a kind of active-response interaction between the merging vehicle and the mainline vehicle in the high-flow bottleneck road section, which expands the division of vehicle lane-changing merging behavior, and the results show that the model can reflect the bidirectional decision-making process of the on-ramp vehicle and the mainline lane rear vehicle and reduce the merging risk of the vehicle effectively. Wu et al.^18^ conducted a comparative analysis of the lane-changing merging behavior of vehicles by using NGSIM trajectory data, and the results showed that factors such as the position and speed of the merging vehicle in the acceleration lane are important in influencing the merging process.

In summary, the vehicle interaction process is a microscopic behavior, but the microscopic behavior will have an impact on the macroscopic traffic flow. Similarly, at the macroscopic level, factors such as vehicle travel location can significantly affect the decision tendency of vehicles to choose to merge. Therefore, the operational dynamics of macro vehicular flow can also affect micro vehicles decisions. Although the above studies can show the macro-micro interaction process of vehicles, the existing studies lack vehicle behavior modeling that combines the macro and micro perspectives. To show and analyze the vehicle interaction characteristics, we analyze the vehicle’s microscopic decision-making behavior with a decision cost function model, and explore the vehicle’s decision-making tendency affected by macroscopic factors based on non-cooperative evolutionary game theory. Based on the phenomenon of vehicle interaction behavior found in the trajectory dataset, this study tries to analyze the consistency of vehicle decision-making under the macro-micro perspective, and then provide theoretical references for related research.

## Vehicle trajectory data analysis

### Trajectory dataset

**Fig. 1:**
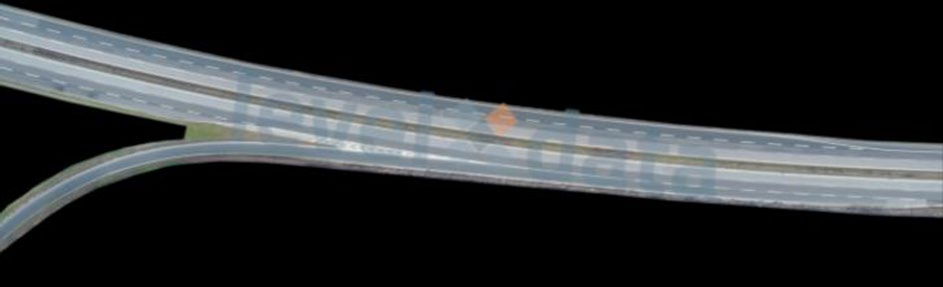
Scenario of the dataset.

The exiD (The exits and entries drone dataset) open dataset for the highway^19^ is based on the lanelet2 high-precision map format to provide a relatively objective reference for the vehicle entry data, which reduces the impact of subjectivity on the study of lane-changing behavior, and the exiD open dataset can be requested and obtained free of charge from the website https://www.exid-dataset.com. Figure 1 shows the image of the vehicle merging roadway provided in the trajectory data. This paper is based on the above trajectory dataset to compare and analyze the interaction behavior and driving characteristics of vehicle lane-changing merging.

### Vehicle interaction behavior analysis

**Fig. 2:**
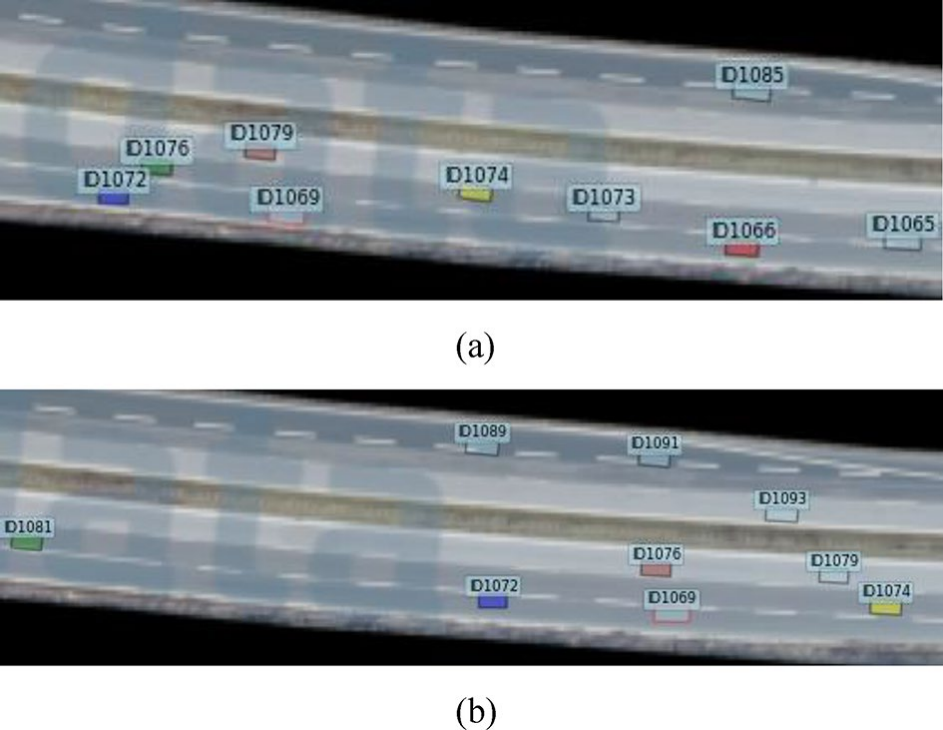
Scenario of the dataset. (**a**) The scene before the mainline rear car change lane and cut out, (**b**) The scene after the mainline rear car change lane and cut out.

Drivers usually need to pre-select the convergence gap and judge the lane change conditions before merging into the mainline lane, and the convergence gap determines the interaction process between vehicles. By screening the trajectory data of vehicles and using the visualization tool, it is found that there exist two other vehicular interaction phenomena, which effectively helped the ramp vehicles to complete the merging process. The visualization tool run_track_visualization.py in the track dataset enables to visualize the vehicle driving process. Figure 2 shows the process of mainline rear car change lane and cut out behavior during time frames between 26,950 and 27,060, the red box is the ramp vehicle ID1069, the yellow vehicle is the left front vehicle ID1074, and the green vehicle is the left rear vehicle ID1076. As vehicle ID1076 changes lane and cuts out to the inside, the left rear vehicle of vehicle ID1069 changes from ID1076 to ID1081, and vehicle ID1069 subsequently change lane to merge.

**Fig. 3:**
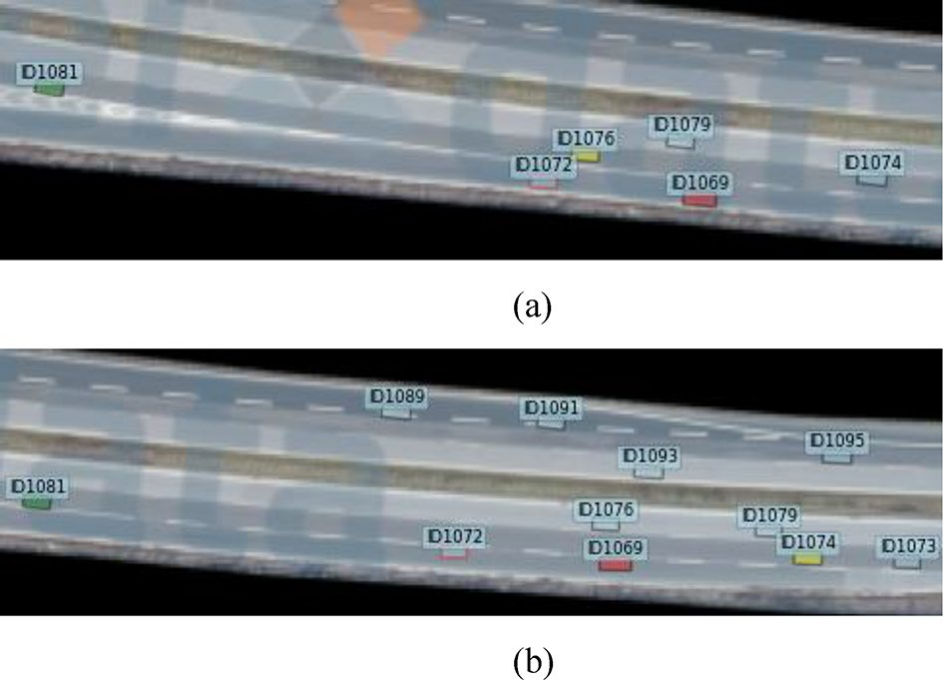
Vehicle merging gap changes. (**a**) The scene before the mainline front car change lane and cut out, (**b**) The scene after the mainline front car change lane and cut out

Figure 3 shows the vehicle interaction process for the front lane change and cut-out of the vehicle, the red box is the ramp vehicle ID1072, the yellow vehicle is the left front vehicle ID1076, and the green vehicle is the left rear vehicle ID1081. when vehicle ID1076 changes lane and cuts out to the inside lane, the left front of vehicle ID1072 changes from ID1076 to ID1074, and vehicle ID1072 subsequently change lane to merge. By combining Figs. 2 and 3, the change in vehicles directly interacting with the ramp vehicle is usually due to the presence of a previous ramp vehicle ahead of the ramp vehicle that needs to merge, or a slow vehicle. The mainline vehicles interacting with the ramp vehicle are staggered, thus accommodating the need for the ramp vehicle to merge and allow it to complete the lane change process.

**Fig. 4:**
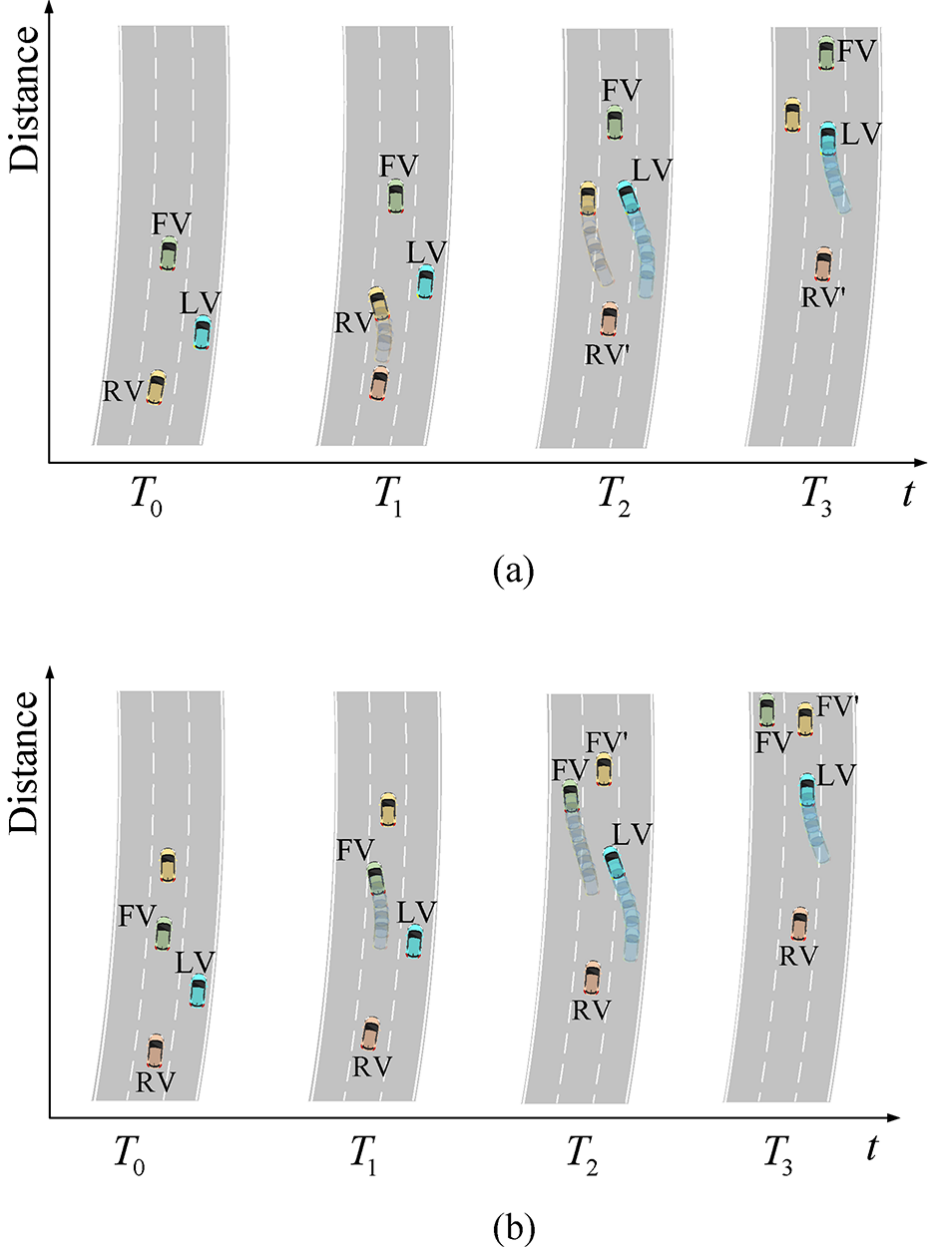
Mainline vehicle lane change cut-out process.(**a**) Process of rear mainline car changing lane and cutting out, (**b**) Process of front mainline car changing lane and cutting out

To illustrate in detail the vehicle interaction behavior in the trajectory dataset, we illustrate the dynamic schematic in Fig. 4. Figure 4a shows the behavioral process of the rear side cut-out of the mainline vehicle and the change of the convergence gap. At the time ${T_0}$, when the merging vehicle LV(ID1069) is in the longitudinal position previously behind the mainline vehicle RV(ID1076) and attempts to change lane to merge, the RV needs to adopt a certain strategy to deal with the merging intention of the LV. In addition to decelerating to give way, the RV performs a lane change and cuts out by judging the condition of the inside lane$\left( {{T_0}\sim {T_2}} \right)$. At this time, the vehicle behind the merging gap changes from the RV to the RV‘(ID1081), and the merging gap changes abruptly, and the LV detects that the RV is moving out of the outside lane and performs a lane change and merging by judging the merging condition again$\left( {{T_1}\sim {T_3}} \right)$. Figure 4b shows that the mainline front FV(ID1076) chooses to change lane and cut out when it is influenced by the vehicle FV’(ID1074) in front of it, and at the same time increases the forward gap of the LV(ID1072), prompting the LV to change lane and converge. In this scenario, the vehicle ID1076 serves as the left rear vehicle of ID1069 and the left front vehicle of ID1072. Since both ramp vehicles and mainline vehicles exist in a continuous traffic flow of travel during a certain cycle time, the front car change lane and cut out behavior that appears in the trajectory dataset is usually accompanied by the rear car change lane and cut out.

**Fig. 5:**
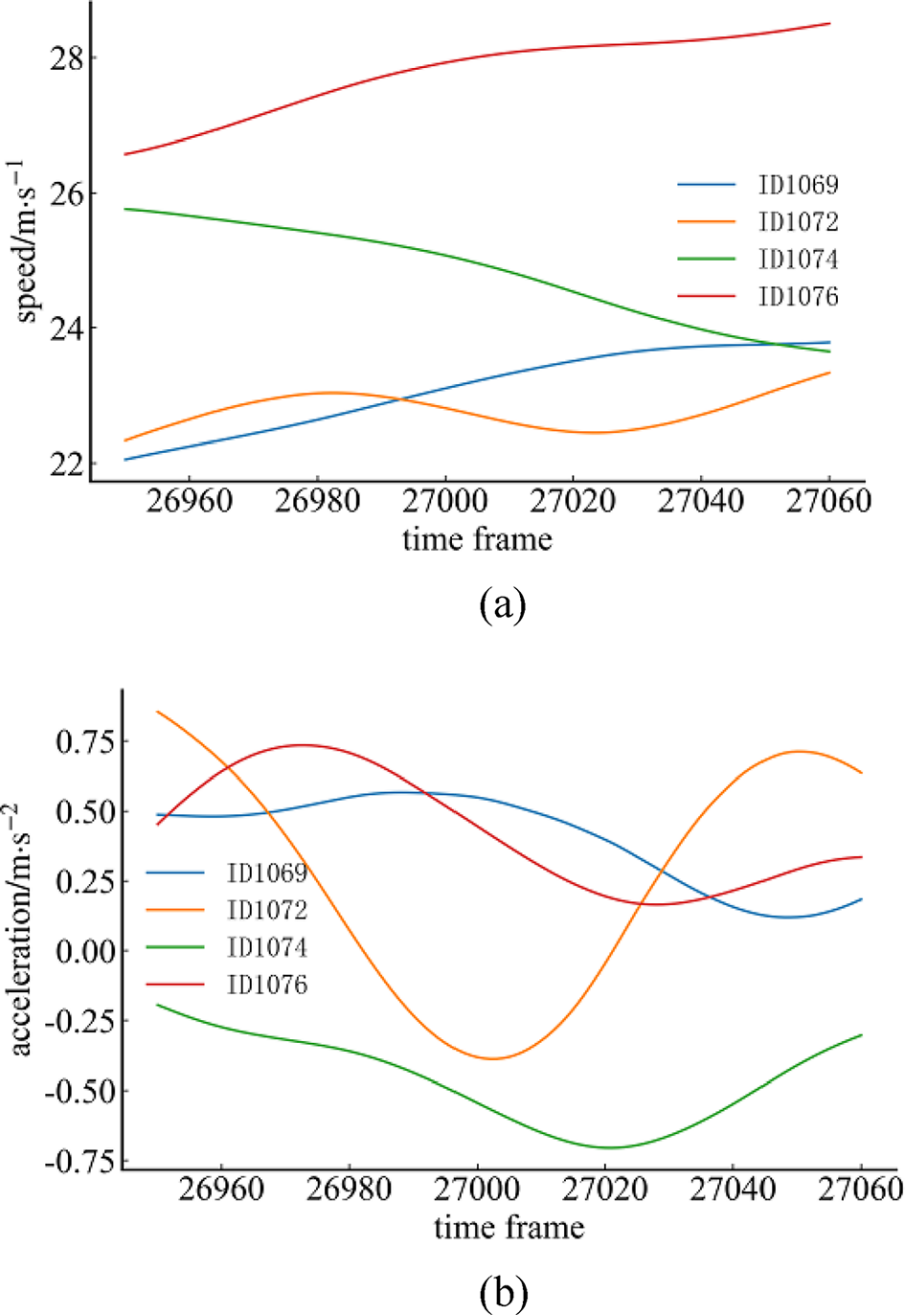
Changes of vehicle speed and acceleration.(**a**) Speed changes during lane change and cut-out of rear mainline vehicle. (**b**) Speed changes during lane change and cut-out of front mainline vehicle.

From the results of the interaction decision, the collaborative behavior of the cut-out vehicle provides a gap for the merging of the ramp vehicle, prompting it to merge smoothly. As shown in Fig.5a, from the viewpoint of the vehicles’ longitudinal speed comparison, the speed of the cut-out vehicle ID1076 is usually higher than that of the vehicle in front and ramp vehicle. Figure 5b demonstrates the longitudinal acceleration of the vehicle, where vehicle ID1076 not only has a high longitudinal travel speed but also a high longitudinal acceleration prior to the vehicle interaction process. This suggests that the vehicle did not demonstrate deceleration yielding inclination in the face of ramp vehicle preparing to merge and lower speed vehicle in front of it, thus providing evidence of its readiness to choose to change lane and cut out to the inside. The subsequent longitudinal acceleration change of the lane-changing and cut-out vehicle in Fig. 5b is due to the lane changing process.

From the perspective of game decision-making and cost analysis of the rear side of the cut-out behavior, When the ramp vehicle explicitly exists in the convergence intention and the speed of the main line back of the vehicle expectations of conflict, the main line back of the vehicle needs to decide on the convergence intention of the judgment or game interaction, the main line vehicle can be with the convergence of the vehicle strategy game, but when the inside vehicle to meet the lane change constraints, the main line vehicle can change lane away from the vehicle to make their expectations of the speed of the gain and ramp vehicles to meet the merging of the willingness. As a result, the rear vehicle on the mainline chooses to change lane to cut out and avoids the direct game with the merging vehicle, which not only reduces the loss of efficiency cost but also reduces the risk of conflict between the two parties traveling intentions at the same time. Similarly, the front vehicle chooses to cut out ahead to reduce its own influence by the vehicle in front of it, and chooses to change lane to the inside lane, at this time, the ramp vehicle needs to merge, and the front vehicle changes lane to cut out to assist the ramp vehicle to merge.

As shown in Table 1 for different vehicle interaction decision types and behavioral results comparison. From the vehicle game interaction results, lane changing and cut out behavior and deceleration to give way both has collaborative interaction behavioral characteristics, is to a certain extent to assist the ramp vehicle merge. Through the comparative analysis of the vehicle interaction process, it is found that the driving state of the mainline vehicle is affected by the intent of the ramp vehicle to merge, and the former chooses the corresponding strategy based on weighing and considering the cost loss corresponding to different decisions. Therefore, the vehicle lane-changing and cutting-out behaviors can’t be analyzed from the perspective of the result of collaboration only, and the decision-making process of the mainline vehicle is analyzed based on the perspective of the cost angle of the cut-out vehicle game, which is found to be the interactive behavior. does not have the subjective will to collaborate, but is a collaborative outcome formed by the mainline vehicles based on not reducing their benefits.

**Table 1:** Comparison of vehicle interaction behavior.

Interaction type	The process of interaction	Mainline vehicle response strategy
Slow down and give way	LV waiting for RV to slow down and give way to merge in	slows down and gives way
change lane and cut out	LV waits for the RV or FV changing lane to cut out and then judges the merging condition again	reducing the loss of speed cost while assisting ramp vehicles to merge

## Model

### Lane change trajectory constraints

It is well known that quintic polynomial curve is usually solved at the cost of a Jerk’s function and through the generalized Euler-Lagrange equation for the lane-changing trajectory, and thus usually satisfies the comfort requirement in lane-changing decisions, assuming that the lane-changing trajectory is the vehicle’s control-of-mass trajectory then the trajectory curve is

1 $$\left\{ {\begin{array}{*{20}{c}} {x\left( t \right)={a_0}{t^5}+{a_1}{t^4}+{a_2}{t^3}+{a_3}{t^2}+{a_4}{t^1}+{a_5}{t^0}} \\ {y\left( t \right)={b_0}{t^5}+{b_1}{t^4}+{b_2}{t^3}+{b_3}{t^2}+{b_4}{t^1}+{b_5}{t^0}} \end{array}} \right.$$

Here, ${a_i}\left( {i=0,1, \ldots ,5} \right)$ and ${b_i}\left( {i=0,1, \ldots ,5} \right)$ are the coefficients of the track change curve.

Based on the vehicle state constraints at the initial moment of lane change and the end moment of lane change, the vehicle longitudinal trajectory, velocity and acceleration profiles can be represented as matrices

2 $$X=\left[ {\begin{array}{*{20}{c}} {{x_0}} \\ {{v_0}} \\ {{a_{lon0}}} \\ {\begin{array}{*{20}{c}} {\begin{array}{*{20}{c}} {{x_1}} \\ {{v_1}} \end{array}} \\ {{a_{lon1}}} \end{array}} \end{array}} \right]=\left[ {\begin{array}{*{20}{c}} {t_{0}^{5}} \\ {5t_{0}^{4}} \\ {20t_{0}^{3}} \\ {\begin{array}{*{20}{c}} {\begin{array}{*{20}{c}} {t_{1}^{5}} \\ {5t_{1}^{4}} \end{array}} \\ {20t_{1}^{3}} \end{array}} \end{array}\begin{array}{*{20}{c}} {t_{0}^{4}} \\ {4t_{0}^{3}} \\ {12t_{0}^{2}} \\ {\begin{array}{*{20}{c}} {\begin{array}{*{20}{c}} {t_{1}^{4}} \\ {4t_{1}^{3}} \end{array}} \\ {12t_{1}^{2}} \end{array}} \end{array}\begin{array}{*{20}{c}} {t_{0}^{3}} \\ {3t_{0}^{2}} \\ {6{t_0}} \\ {\begin{array}{*{20}{c}} {\begin{array}{*{20}{c}} {t_{1}^{3}} \\ {3t_{1}^{2}} \end{array}} \\ {6{t_0}} \end{array}} \end{array}\begin{array}{*{20}{c}} {t_{0}^{2}} \\ {2t_{0}^{1}} \\ 2 \\ {\begin{array}{*{20}{c}} {\begin{array}{*{20}{c}} {t_{1}^{2}} \\ {2{t_1}} \end{array}} \\ 2 \end{array}} \end{array}\begin{array}{*{20}{c}} {t_{0}^{1}} \\ 1 \\ 0 \\ {\begin{array}{*{20}{c}} {\begin{array}{*{20}{c}} {t_{1}^{1}} \\ 1 \end{array}} \\ 0 \end{array}} \end{array}\begin{array}{*{20}{c}} {t_{0}^{0}} \\ 0 \\ 0 \\ {\begin{array}{*{20}{c}} {\begin{array}{*{20}{c}} {t_{1}^{0}} \\ 0 \end{array}} \\ 0 \end{array}} \end{array}} \right]\left[ {\begin{array}{*{20}{c}} {{a_5}} \\ {{a_4}} \\ {{a_3}} \\ {\begin{array}{*{20}{c}} {\begin{array}{*{20}{c}} {{a_2}} \\ {{a_1}} \end{array}} \\ {{a_0}} \end{array}} \end{array}} \right]=T \times A$$

Similarly, the matrix of lateral trajectory, velocity, and acceleration profiles of the vehicle can be expressed as

3 $$Y={\left[ {0,0,0,W,0,0} \right]^T}=T \times {\left[ {{b_5},{b_4},{b_3},{b_2},{b_1},{b_0}} \right]^T}$$

Here, ${x_0}$ is the longitudinal position of the vehicle at the initial moment. ${v_0}$ and ${v_1}$are the longitudinal velocity of the vehicle at the initial and final moments. ${a_{lon0}}$ and ${a_{lon1}}$ are the longitudinal acceleration at the initial and final moments. and* W* is the lateral offset.

### Following distance and lane change gap constraints

Vehicles need to satisfy the constraints of safe following distance in the longitudinal direction, this paper adopts the cooperative adaptive cruise control, i.e., CACC model, for portraying the longitudinal safety distance relationship during vehicle traveling.

4 $$\left\{ {\begin{array}{*{20}{c}} {{v_i}={v_{pre,i}}+{k_p}e+{k_d}\mathop e\limits^{ \bullet } } \\ {e={d_{cur}} - {d_{min}} - {L_{car}} - {t_{hw}}{v_i}} \end{array}} \right.$$

Here,${v_i}$ is the speed of the vehicle at the current moment;${v_{pre,i}}$ is the speed of the vehicle at the previous moment;* e*is the vehicle spacing error term; ${d_{cur}}$is the headway;${d_{min}}$ is the minimum stopping distance;${L_{car}}$is the body length; ${t_{hw}}$ is the safe headway.

**Fig. 6:**
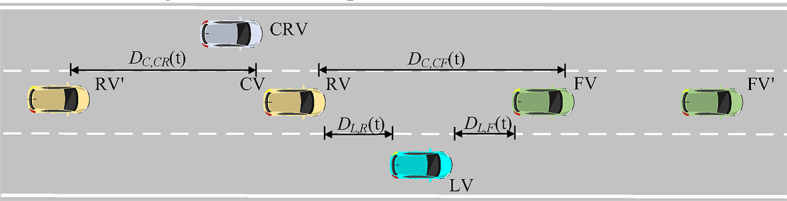
Distance between vehicles constraints.

The lane-changing process of a vehicle usually includes the generation of lane-changing intention, searching for the lane-changing gap, executing the lane-changing process, and following the vehicle in the target lane. As shown in Fig. 6, For the vehicles with the existence of a clear lane-changing intention, it is necessary to judge the constraints of the convergence gap to execute the lane-changing convergence strategy, and the convergence gap acts as an intermediate link constraining the vehicle’s decision-making and behavioral process.

Based on the vehicle lane-changing safe distance method^20^, the safe lane-changing distance constraints of the merging vehicle and the cut-out vehicle are expressed as

5 $${D_{i,j}}(t) \geqslant {F_{i,j}}(t){\text{ }}$$

6 $${F_{i,j}}(t)={v_i}(t){T_{hw}}+\frac{{v_{i}^{2}(t)}}{{2{a_{i,dec}}(t)}} - \frac{{v_{j}^{2}(t)}}{{2{a_{j,maxdec}}}}$$

7 $${a_{i,dec}}(t)={a_{i,mindec}}+\frac{{{v_i}(t)}}{{{v_{i,max}}(t)}}({a_{i,maxdec}} - {a_{i,mindec}})$$

Here, ${D_{i,j}}(t)$ and ${F_{i,j}}{\text{(}}t{\text{)}}$ are the longitudinal inter-vehicle distance and safety distance requirements of vehicle* i* and vehicle* j* respectively. ${T_{hw}}$ is the desired headway. ${v_i}(t)$ and ${v_j}(t)$ are the longitudinal speeds of the vehicle respectively. ${a_{i,dec}}(t)$ is the deceleration of the vehicle* i* at the time* t*. ${a_{i,mindec}}$ and ${a_{i,maxdec}}$ are the minimum and maximum deceleration of the vehicle* i* respectively. and ${v_{i,max}}(t)$ is the maximum traveling speed of the vehicle* i*.

### Gain from the game decision to merge vehicles

Evolutionary game theory is widely used in the study of decision-making problems such as vehicle lane changing. Its theoretical idea is to adaptively adjust the strategies and choose the strategy with high returns through continuous evolution, which is usually regarded as an evolutionary process with the concept of time, and the evolutionary game theory describes the trend of the evolution of different strategies through the dynamic equation of evolution. Evolutionary game theory explains the decision-making tendency of a single individual from the perspective of a group, and when the group is in different stages, individuals will choose behaviors that conform to the evolutionary direction of the group to maximize their benefits, thus revealing the changes in the direction of the stable evolution of the game strategy in different environments.

For ramp vehicles, choosing the appropriate merging gap can shorten the merging time and reduce the risk of vehicle collision, the process of on-ramp vehicles choosing this gap is to improve their gains. This paper portrays the decision-making process of the vehicles through a non-cooperative evolutionary game model. The decision benefits of remittance and mainline vehicles are expressed as follows

8 $$L=\mu Le+\left( {1 - \mu } \right)Ls{\text{ }}$$

9 $$M=\rho Me+\left( {1 - \rho } \right){M_s}$$

Here, $Le$ and $Ls$ are the efficiency gains and safety gains for merging vehicle, respectively;$Me$and$M{\text{s}}$are the efficiency gains and safety gains for mainline vehicle, respectively; $\mu$and$\rho$are the respective efficiency weighting factors.

The safety of merging and mainline vehicles is affected by vehicle spacing and lane change demand distance, expressing the safety gain of both as

10 $${L_s}={L_s}=\frac{{\Delta S - {S_{min}}}}{{{S_{max}} - {S_{min}}}}$$

11 $${S_{min}} \geqslant {F_{i,j}}(t){\text{ }}$$

Here, $\Delta S$is the longitudinal spacing of vehicle at the moment of decision-making. ${S_{max}}$ is the maximum distance between cars that need to change lane by gaming. ${S_{min}}$ is the minimum distance between cars that can change lane by slowing down with the assistance of the rear car.

The local path driving behavior of an autonomous vehicle can be triggered by a scenario, in this paper, we discretize the driving task of the vehicle, the driving task of the ramp vehicle is to game with the mainline vehicle and change lane to the outer lane of the mainline, and the driving task of the mainline vehicle is to drive out of the converging road section smoothly at the desired driving speed. When the merging vehicle changes lane to the mainline lane, it usually increases the travel time of the mainline vehicle, and when the ramp vehicle is not gaming with the mainline vehicle, the latter’s travel time is less affected by the gaming process. Therefore, the efficiency gains of the vehicles are expressed separately as

12 $${L_e}=\frac{{{t_L} - {t_{Lmin}}}}{{{t_{Lmax}} - {t_{Lmin}}}}$$

13 $$Me=\frac{{{t_M} - {t_{Mmin}}}}{{{t_{Mmax}} - {t_{Mmin}}}}$$

Here, ${t_L}$ is the time to change lane for the different decisions of the converging vehicle; ${t_{Lmax}}$ and ${t_{Lmin}}$ are the maximum and desired traveling time considering the lane change process of the converging vehicle, respectively. ${t_M}$ is the time for the mainline vehicle to pass the confluence section by making different decisions. ${t_{Mmax}}$ and ${t_{Mmin}}$ are the maximum and desired travel times for the mainline vehicle to pass the confluence section by considering the influence of the game process with the ramp vehicle, and the desired travel time is the time required for the vehicle to exit the confluence section under the speed of the free traffic flow.

Considering the future development of vehicles, this paper establishes a common unified driving weight function for the vehicle model, sets the vehicle to adjust the efficiency and safety gain weights according to the traffic environment. The closer the ramp vehicle location is to the end of the acceleration lane the more the vehicle expects to complete the lane change convergence process. Therefore, the weights of the converging vehicles on the efficiency gains are expressed as respectively:

14 $$\mu ={\text{max}}\left\{ {{\text{min}}\left\{ {\frac{{S - {S_{\hbox{min} }}}}{{{S_{{\text{max}}}} - {S_{\hbox{min} }}+\eta }},0.7} \right\},0.3} \right\}{\text{ }}$$

Here,* S*is the lane change merge position corresponding to the decision moment of the ramp merging vehicle; ${S_{\hbox{min} }}$is the minimum desired merge position of the merging vehicle in the acceleration lane; ${S_{\hbox{max} }}$is the furthest merge position of the merging vehicle in the acceleration lane; and $\eta$ is the smaller value that ensures that the denominator is not zero.

Combining the gains of the above vehicles, represents the gains from gaming vehicles as shown in Table 2.

**Table 2:** Gains matrix.

	MV		
		Give way* y*	Not give way 1-*y *
LV	Merging* x*	$$\left[ \begin{gathered} \mu Le + \left( {1 - \mu } \right)Ls{\text{ }}, \hfill \\ - \rho Me + \left( {1 - \rho } \right)Ms \hfill \\ \end{gathered} \right]$$	$$\left[ \begin{gathered} - \mu Le - \left( {1 - \mu } \right)Ls{\text{ }}, \hfill \\ - \rho Me - \left( {1 - \rho } \right)Ms \hfill \\ \end{gathered} \right]$$
	Not merging 1-*x*	$$\left[ \begin{gathered} - \mu Le + \left( {1 - \mu } \right)Ls{\text{ }}, \hfill \\ - \rho Me + \left( {1 - \rho } \right)Ms \hfill \\ \end{gathered} \right]$$	$$\left[ \begin{gathered} - \mu Le + \left( {1 - \mu } \right)Ls{\text{ }}, \hfill \\ \rho Me + \left( {1 - \rho } \right)Ms \hfill \\ \end{gathered} \right]$$

The expected payoff for the decision of an incoming vehicle to change lane is given by

15 $${E_{L1}}=2\left( {\mu Le+\left( {1 - \mu } \right)Ls} \right)y+\left( { - \mu Le - \left( {1 - \mu } \right)Ls} \right)$$

The expected payoff for the decision of a merging vehicle not to change lane is given by

16 $${E_{L2}}= - \mu Le+\left( {1 - \mu } \right)Ls$$

The expected return of a remitting vehicle choosing to remit with probability* x* and choosing not to remit with probability $1 - x$ is

17 $$\begin{gathered} {E_L}=2\left( {\mu Le+\left( {1 - \mu } \right)Ls} \right)xy - 2\left( {1 - \mu } \right)Lsx \hfill \\ {\text{ }} - \mu Le+\left( {1 - \mu } \right)Ls \hfill \\ \end{gathered}$$

The evolutionary dynamic equation for the merging vehicle selection merging is

18 $${F_L}=\frac{{\partial x}}{{\partial t}}=2x\left( {1 - x} \right)\left[ {\left( {\mu Le+\left( {1 - \mu } \right)Ls} \right)y - \left( {1 - \mu } \right)Ls} \right]$$

An evolutionary dynamic equation is developed for the mainline vehicle with continuous decision time, and the expected return corresponding to its choice of give-way strategy is

19 $${E_{M1}}= - \rho Me+\left( {1 - \rho } \right)Ms$$

The expected gain corresponding to a vehicle behind the mainline choosing the strategy of not giving way is

20 $${E_{M2}}= - 2\left( {\rho Me+\left( {1 - \rho } \right)Ms} \right)x+\left( {\rho Me+\left( {1 - \rho } \right)Ms} \right)$$

The expected payoff for a mainline vehicle choosing to yield with probability* y* and not yielding with probability $1 - y$ is

21 $$\begin{gathered} {E_M}= - 2\rho Mey - 2\left( {\rho Me+\left( {1 - \rho } \right)Ms} \right)x \hfill \\ {\text{ }}+2\left( {\rho Me+\left( {1 - \rho } \right)Ms} \right)xy+\left( {\rho Me+\left( {1 - \rho } \right)Ms} \right) \hfill \\ \end{gathered}$$

The evolutionary dynamic equation for the mainline vehicle selection yielding is

22 $${F_M}=\frac{{\partial y}}{{\partial t}}=2y\left( {1 - y} \right)\left[ { - \rho Me+\left( {\rho Me+\left( {1 - \rho } \right)Ms} \right)x} \right]$$

The results of the strategy regarding the choice of merging vehicles and the choice of mainline vehicles to give way are obtained through an Eq. (23).

23 $$\frac{{\partial x}}{{\partial t}} = 0,{\text{ }}\frac{{\partial y}}{{\partial t}} = 0,x,y \in \left[ {0,1} \right]$$

By solving Eq. (23), four sets of solutions (0,0), (0,1), (1,0) and (1,1) can be obtained. The corresponding combinations of strategies are (not merge, not yield), (not merge, yield), (merge, not yield) and (merge, yield). In addition to the above four sets of differential equation solutions, the system of differential equations has a saddle point $(x*,y*)$ with an uncertain direction of convergence. In the yielding strategy, mainline vehicles choose to slow down and yield or cut out based on lane conditions and decision costs, and this part of the analysis will be modeled in the next section. Denote the Jacobi matrix of the decision solution of the game system as

24 $${J_{UM}}=\left( {\begin{array}{*{20}{c}} {\frac{{\partial {F_I}}}{{\partial x}}}&{\frac{{\partial {F_I}}}{{\partial y}}} \\ {\frac{{\partial {F_R}}}{{\partial x}}}&{\frac{{\partial {F_R}}}{{\partial x}}} \end{array}} \right)=\left( {\begin{array}{*{20}{c}} {2\left( {1 - 2x} \right) \times \left[ {\left( {\mu Le+\left( {1 - \mu } \right)Ls} \right)y - \left( {1 - \mu } \right)Ls} \right]}&{2x\left( {1 - x} \right)\left( {\mu Ie+\left( {1 - \mu } \right)Is} \right)} \\ {2y\left( {1 - y} \right)\left( {\rho Me+\left( {1 - \rho } \right)Ms} \right)}&{2\left( {1 - 2y} \right) \times \left[ { - \rho Re+\left( {\rho Re+\left( {1 - \rho } \right)Rs} \right)x} \right]} \end{array}} \right)$$

The $\left| {{J_{IR}}} \right|$ and $tr\left( {{J_{IR}}} \right)$ of this matrix are obtained as

25 $$\begin{gathered} \left| {{J_{IM}}} \right|=\left[ {2\left( {1 - 2x} \right)\left( {\left( {\mu Le+\left( {1 - \mu } \right)Ls} \right)y - \left( {1 - \mu } \right)Ls} \right)} \right] \times \left[ {2\left( {1 - 2y} \right)\left( { - \rho Me+\left( {\rho Me+\left( {1 - \rho } \right)Ms} \right)x} \right)} \right] \hfill \\ {\text{ }} - \left[ {2y\left( {1 - y} \right)\left( {\rho Me+\left( {1 - \rho } \right)Ms} \right)} \right] \times \left[ {2x\left( {1 - x} \right)\left( {\mu Le+\left( {1 - \mu } \right)Ls} \right)} \right] \hfill \\ \end{gathered}$$

26 $$tr\left( {{J_{IM}}} \right)=\left[ {2\left( {1 - 2x} \right)\left( {\left( {\mu Le+\left( {1 - \mu } \right)Ls} \right)y - \left( {1 - \mu } \right)Ls} \right)} \right]+\left[ {2\left( {1 - 2y} \right)\left( { - \rho Me+\left( {\rho Me+\left( {1 - \rho } \right)Ms} \right)x} \right)} \right]$$

The stability of the equilibrium points was analyzed based on the Lyapunov stability principle, and the results are shown in Table 3.

**Table 3:** Stability analysis of equilibrium points.

equilibrium point	evolutionarily stable strategy	strategy combination
(0,0)	stable point	(not merge, not yield)
(0,1)	unstable point	(not merge, yield)
(1,0)	unstable point	(merge, not yield)
(1,1)	stable point	(merge, yield)
$(x*,y*)$	saddle point	—

### Cost of gaming decisions for mainline vehicles

Although the ramp vehicle chooses the optimal convergence gap and the mainline vehicle can only make a give-way strategy according to the evolutionary game results, by analyzing the vehicle interaction characteristics in the trajectory dataset, we find that some of the mainline vehicles reduce the impact of the ramp vehicle’s merging behavior on itself by cut out to the inside lane. Through the lane choice cost function above, we provide a complementary coping strategy for the mainline vehicle when the mainline vehicle becomes the object of the game of the optimal convergence gap, it can choose the driving lane optimally by judging the lane switching cut-out condition and lane choice cost. When the mainline vehicle becomes the object of the game of optimal merging gap, it can choose the traveling lane by judging the lane change and cut-out condition and the lane choice cost of the inside lane, if the lane change cut-out cost is smaller, the mainline vehicle does not need to continue to decelerate to provide convergence gap for the on-ramp vehicle, but it can assist the on-ramp vehicle to complete the process of convergence by changing lane to provide a merging gap for the latter, which can be achieved without increasing the cost of traveling for the mainline vehicle.

Mainline vehicles need to minimize their driving costs, including safety and efficiency costs when considering the presence of ramp vehicles with clear intent to merge. In this paper, we depict the decision-making process of the mainline vehicle’s judgment of the vehicle through a decision cost function. Based on the non-cooperative game^21,22^ and Nash equilibrium methods^23^ to solve the lane change cut-out and its decision propensity of the mainline vehicles, the vehicle traveling efficiency cost is usually considered as the difference between the current longitudinal traveling speed of the vehicle and the desired speed of the lane, which defines the speed cost function of the vehicle as

27 $${J_{eff}}(t)={e^{\left( {{v_{cur}}(t) - {v_{n,max}}(t)} \right)}},{\text{ }}n \in \left\{ {0,1} \right\}$$

28 $${v_{n,max}}(t)=\left\{ {\begin{array}{*{20}{c}} {min({v_{n,fcar}}(t),{v_{mercar}}(t)),{\text{ }}n=0} \\ {min(v{}_{{n,lim}},{v_{n,fcar}}(t)),{\text{ }}n=1} \end{array}} \right.$$

Here, ${v_{cur}}(t)$ is the longitudinal traveling speed of the current decision-making vehicle at the time* t*. ${v_{n,max}}$ is the maximum desired traveling speed of the* n* lane,* n* is 0 for the outer lane of the main line, and* n* is 1 for the inner lane of the main line. ${v_{n,fcar}}(t)$ and ${v_{mercar}}(t)$ are the traveling speeds of the vehicle in front of the nth lane and the converging vehicle at the time* t*, respectively. and $v{}_{{n,lim}}$ is the maximum traveling speed of the* n* lane.

The safety cost of a vehicle changing lane is related to the relative position and relative speed of the vehicle, reflecting the risky conflict with the interacting vehicle at some time in the future, defining the safety cost function of the vehicle as

29 $${J_{sec}}(t)=n{J_{lats}}(t)+{\left( {n - 1} \right)^2}{J_{lons}}(t){\text{ }},{\text{ }}n \in \left\{ {0,1} \right\}$$

30 $${J_{lons}}(t)={\delta _{lon,\Delta v}}{\left( {\Delta {v_{lon,ij}}(t)} \right)^2}\varepsilon (\Delta {v_{lon,ij}}(t))+\frac{{{\delta _{lon,\Delta s}}}}{{{{\left( {\Delta {s_{lon,ij}}(t)} \right)}^2}+\xi }}$$

31 $$\varepsilon (\Delta {v_{lon,ij}}(t))=\left\{ {\begin{array}{*{20}{c}} {0{\text{ }},{\text{ }}\Delta {v_{lon,ij}}(t)<0} \\ {1{\text{ , }}\Delta {v_{lon,ij}}(t)>0} \end{array}} \right.$$

32 $$\Delta {v_{lon,ij}}(t)={v_i}(t) - {v_j}(t)$$

33 $${J_{lats}}(t)={\delta _{lat,\Delta v}}{\left( {\Delta {v_{lat,ik}}(t)} \right)^2}\varepsilon (\Delta {v_{lat,ki}}(t))+\frac{{{\delta _{lat,\Delta s}}}}{{{{\left( {\Delta {s_{lat,ki}}(t)} \right)}^2}+\xi }}$$

34 $$\varepsilon (\Delta {v_{lat,ki}}(t))=\left\{ {\begin{array}{*{20}{c}} {0{\text{ }},{\text{ }}\Delta {v_{lat,ki}}(t)<0} \\ {1{\text{ , }}\Delta {v_{lat,ki}}(t)>0} \end{array}} \right.$$

35 $$\Delta {v_{lon,ki}}(t)={v_k}(t) - {v_i}(t)$$

Here, ${J_{lons}}(t)$ is the safety cost when a vehicle*i* chooses to drive in the current lane, taking into account the relative distance $\Delta {s_{lon,ij}}(t)$ and speed difference $\Delta {v_{lon,ij}}(t)$ with the converging vehicle* j*. ${v_i}(t)$ and ${v_j}(t)$ are the speeds of the vehicle* i* and vehicle* j* at the time* t*, respectively. ${J_{lats}}(t)$ is the safety cost when a vehicle* i* chooses to change lane and cut out with the vehicle* k* of the inside lane, taking into account the relative distance $\Delta {s_{lat,ki}}(t)$ and the speed difference with the vehicle $\Delta {v_{lat,ki}}(t)$. ${v_k}(t)$ is the speed of the vehicle* k* at the time. $\varepsilon (\Delta {v_{lon,ij}}(t))$ and $\varepsilon (\Delta {v_{lat,ki}}(t))$ are the step functions, considering the effect of the speed difference only if ${\text{ }}\Delta {v_{lon,ij}}(t)>0$ or $\Delta {v_{lat,ik}}(t)>0$.

The total cost function ${J_{total}}$ for constructing the vehicle game decision is

36 $${J_{total}}\left( {\Delta {s_{n,ij}},\Delta {v_{n,ij}},n,t} \right)={\omega _{eff}}\frac{{{J_{eff}}(t)}}{{{J_{effmax}}}}+\left( {1 - {\omega _{eff}}} \right)\frac{{{J_{sec}}(t)}}{{{J_{secmax}}}}$$

Here, ${\omega _{eff}}$ is the coefficient of efficiency cost ${J_{eff}}(t)$in the vehicle decision-making process; ${J_{sec}}(t)$ is the cost of safety in vehicle traveling; ${J_{effmax}}$ and the normalized values of efficiency and safety in the cost function, respectively.

In the vehicle game decision-making interaction process, by considering the safety and efficiency costs of vehicles to minimize the cost function, the vehicle game strategy choice is obtained as

37 $$n(t)=argmin {J_{total}}\left( {\Delta {s_{n,ij}},\Delta {v_{n,ij}},n,t} \right){\text{, }}n \in \left\{ {0,1} \right\}$$

Vehicles choose response strategies optimally by making judgments about driving efficiency and safety costs, and vehicles need to satisfy the range of variables in the gaming decision and lane-changing process.

38 $${v_{min}} \leqslant v(t) \leqslant {v_{max}}$$

39 $${a_{min}} \leqslant a(t) \leqslant {a_{max}}$$

40 $${d_{max}} \leqslant d(t) \leqslant {d_{min}}$$

41 $${W_{min}} \leqslant W \leqslant {W_{max}}$$

42 $${t_{min}} \leqslant {t_1} - {t_0} \leqslant {t_{max}}$$

Here, ${v_{min}}$,${v_{max}}$,${a_{min}}$,${a_{max}}$,${d_{min}}$,${d_{max}}$,${W_{min}}$,${W_{max}}$,${t_{min}}$,${t_{max}}$ are the minimum and maximum speeds, acceleration, deceleration, lateral displacement, and lane change time of the vehicle, respectively.

### The process of vehicle speed matching

In the ramp vehicle speed matching merging gap, the mainline vehicle continues to drive, the ramp vehicle needs to drive to the mainline rear vehicle right in front of a certain distance, to complete the merging gap matching process, the speed matching process is expressed as

43 $${x_{M0}}+\int_{0}^{{\Delta t}} {{v_M}(t)} dt+{D_{L,M}}(t) \leqslant {x_{L0}}+\int_{0}^{{\Delta t}} {{v_L}(t)} dt$$

44 $${v_L}(t)={v_{L0}}+\int_{0}^{t} {{a_L}(\tau )} d\tau$$

Here, are the initial longitudinal positions of the vehicle MV and LV, respectively. ${v_M}(t)$ and ${v_L}(t)$are the velocities of the vehicles MV and LV. ${D_{L,M}}(t)$ is the vehicle merging gap requirement distance. ${v_{L0}}$ is the initial velocity of the vehicle LV. ${a_L}(\tau )$ is the acceleration of the vehicle LV.

## Simulation test and result analysis

To verify the validity of the change lane and cut out model constructed in this paper, simulation tests were carried out using SUMO software, and the model parameters as shown in Table 4. were obtained from the trajectory dataset and literature results^24^.

**Table 4:** Parameter calibration results.

symbolic	symbolic meaning	value
${a_{max}}$	maximum acceleration$/m \cdot {s^{ - 2}}$	3.15
${d_{comf}}$	Comfort Deceleration$/m \cdot {s^{ - 2}}$	1.42
${v_{mlane}}$	Mainline vehicle speed$/m \cdot s$	24.40
${L_{acc}}$	Length of acceleration lane$/m$	200.25
${d_{min}}$	Minimum Parking Distance$/m$	7.47
${L_{car}}$	Vehicle length$/m$	4.70
${k_p}$	Distance between vehicles factor	0.45
${k_d}$	Coefficient of variation of vehicle distance	0.25

**Fig. 7:**
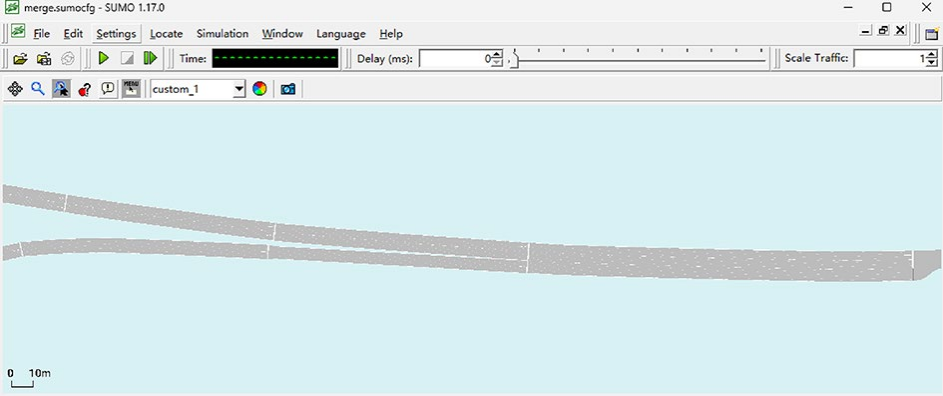
The scenario of traffic flow simulation.

SUMO is an open-source traffic flow simulation software, built-in a variety of vehicle lane-changing and following models and can be developed twice, which usually meets the simulation needs in the field of transportation. Therefore, we choose to use SUMO (version 1.17.0) software as a simulation tool to validate the model in this paper. As shown in Fig. 7, we plotted the lane lines in SUMO using the map dimensions provided in the trajectory dataset as a reference. To verify the validity of the model in this paper, we chose to use the SL2015 model built in SUMO software as a comparison, which is a sub-lane changing model that can demonstrate the continuous lane changing process of vehicles. At the same time, we use the model of this paper and the vehicle convergence data in the trajectory dataset as a reference and analyze the data features present in the dataset.

### Sensitivity analysis

Vehicle speed is the main factor that constitutes the vehicle decision-making cost, at the same time, to portray the real scene in the upstream vehicle traveling state, the simulation test process through a certain proportion of the vehicle from the upstream into the vehicle to set up a different initial speed, through the control of the other variables remain unchanged and select the simulation test scenario in merging vehicle and the inner lane of the speed of the vehicle for the 23.52 m/s and 31.90 m/s, respectively. Adjust the decision-making mainline vehicle speed to 25 ~ 30 m/s, through numerical simulation to analyze the impact of different driving speeds of the mainline vehicle on the decision-making results.

**Fig. 8:**
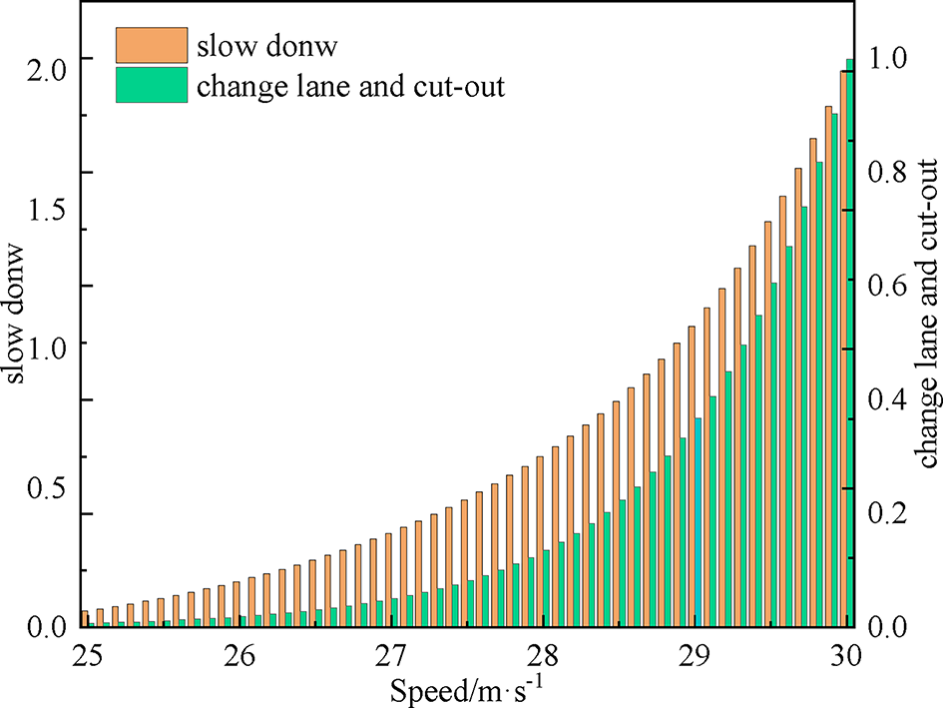
Decision costs of different travel speeds.

As shown in Fig. 8, when the inside lane satisfies the lane change and cut-out conditions, the cost of the mainline vehicle choosing to change lane and cut out ranges between 0 and 1, while the cost of choosing to slow down and yield decision ranges between 0 and 2. When the speed of the mainline vehicles increases, the cost of deceleration yielding and cut-out decisions increases significantly, and the slope of the former curve is larger than the latter, indicating that when the speed of the mainline vehicles is high, the merging behavior of the ramp vehicles increases the speed cost of the mainline vehicles significantly. It is well known that when the inside lane does not satisfy the conditions, the vehicle choosing to change lane and cut out will face safety risks, and the cost of decelerating and yielding is smaller at this time.

**Fig. 9:**
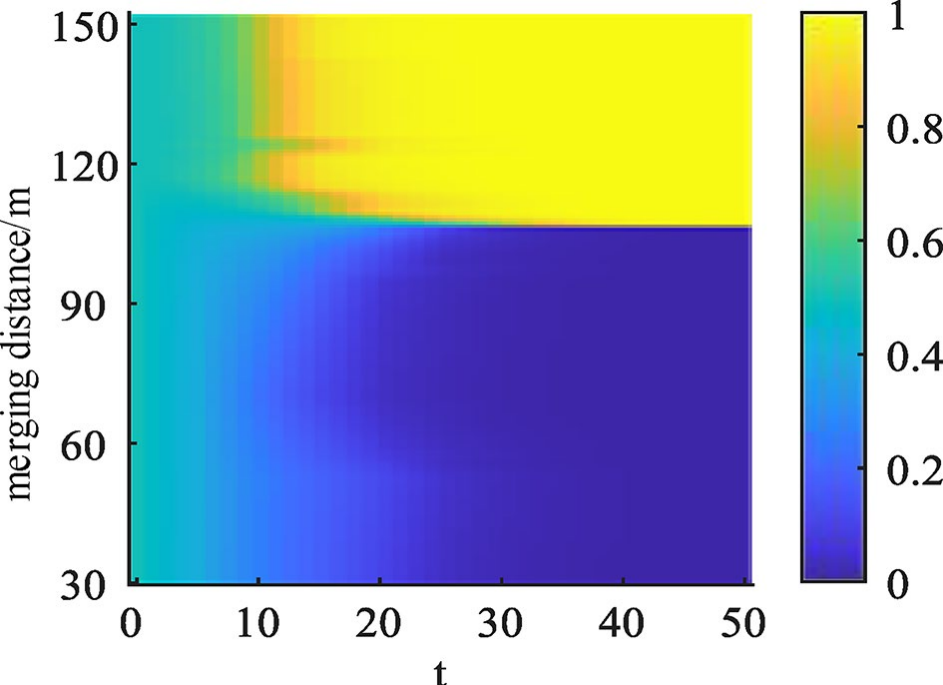
Impact of different locations on merging strategy.

**Fig. 10:**
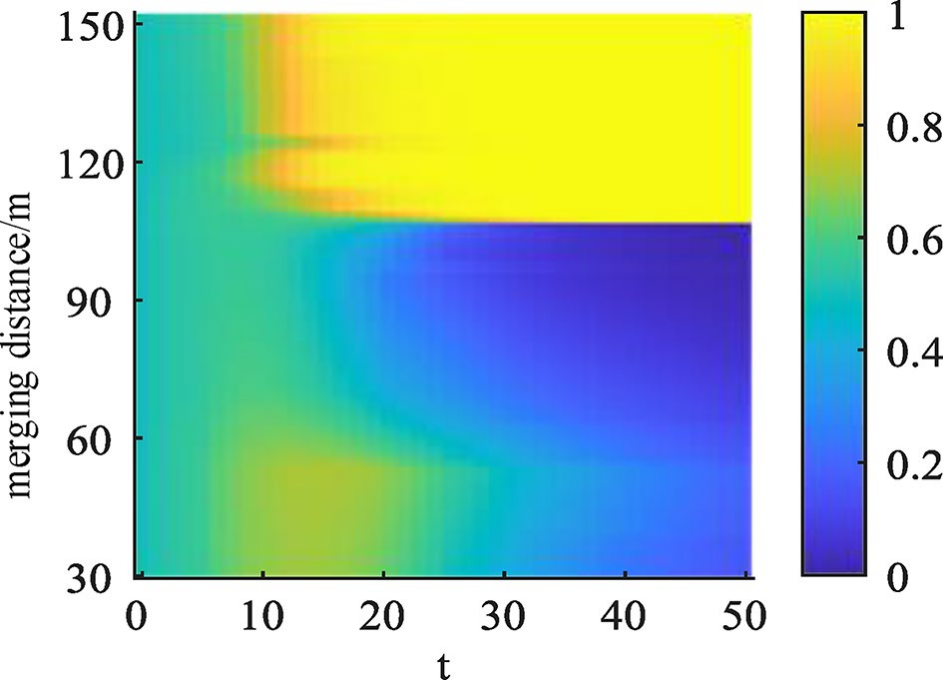
Impact of different locations on giving way strategy.

By adjusting the merging position of the ramp vehicles to explore the stable convergence direction of the vehicles from different positions for the game interaction, as can be seen from Figs. 9 and 10. Through the statistics of the vehicle traveling data to get the distribution of the merging position of the ramp vehicles, the selected vehicle position range between 30 ~ 150 m, when the merging position from 30 m to increase. The ramp vehicles and the mainline vehicles of the stable convergence of the strategy direction of the convergence of the non-convergence and the non-allowance of the road, indicating that according to the driving to the mainline vehicles are more inclined to choose the non-allowance of the road and the ramp vehicles have no advantage of changing lane to merge. With the further increase of the position, it can be found that the stable strategies of the game gradually evolve to give way and merge, indicating that the optimal strategy combinations of both sides are (yield and merge) over a considerable distance and subsequent period.

### Comparison of vehicle merging distances

**Fig. 11:**
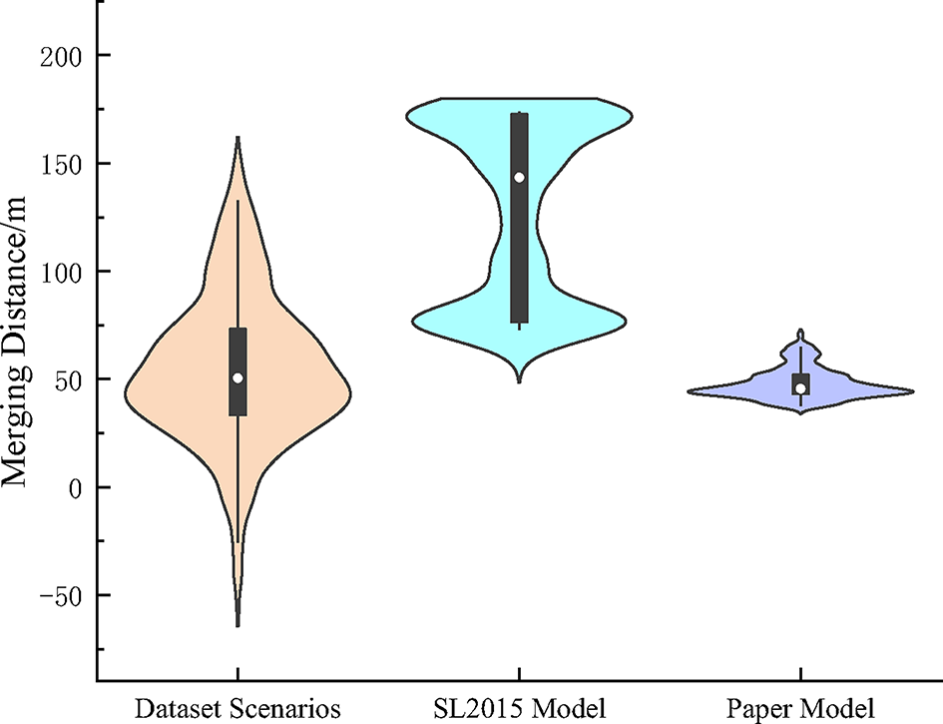
Comparison of vehicles merging distance.

As shown in Fig. 11, the plot of the distribution of the lane change convergence distance is calculated by the merging distance of vehicles in the dataset and the simulation test, using the position of the intersection of the solid and dashed lines on the left side of the acceleration lane in the merging section as the origin. The statistical data show that the merging distance in the dataset scenarios is between the range of [-40.38 m,137.30 m], with the mean value of 57.41 m, standard deviation of 31.76 m. Some of the merging distances in the dataset are less than or converge to 0 because of the vehicles on the ramps in the solid area driving into the main lane. The larger values of lane change merging distance reflect the vehicle in a longer period can’t meet the conditions of convergence and forced to drive to the end of the acceleration lane for convergence, through the data visualization tool found that the situation often occurs in the main line vehicle is not timely and effective to assist the ramp vehicle convergence, forcing the ramp vehicle to drive close to the end of the lane.

Compared with the model and dataset results in this paper, the vehicle merging distance based on the SL2015 model is significantly higher. The reason is because the model does not sufficiently consider the interaction process between vehicles and the vehicles cannot sufficiently collaborate with each other, so the ramp vehicles usually have difficulty in completing the convergence process with a shorter distance. As a result, the distribution of vehicle merging data is concentrated in two locations, larger and smaller. The data distribution results in the Fig. 11 also support the result that vehicles in the SL2015 model usually need to merge at the end of the ramp if they do not complete the merge at the initial position of the ramp.

### Comparative analysis of vehicle merging security

**Fig. 12:**
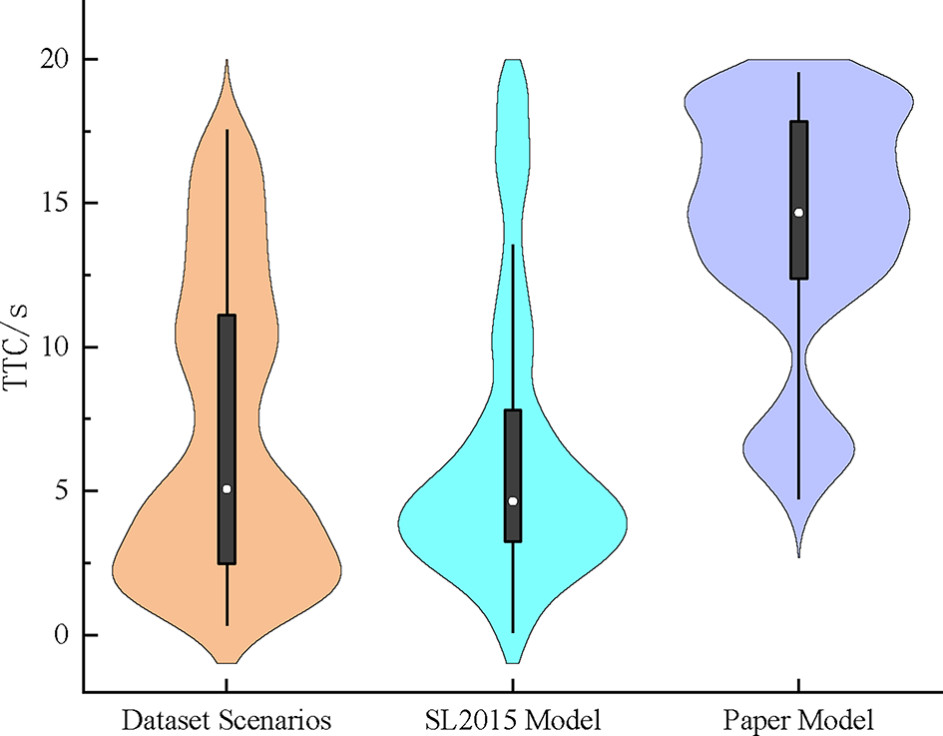
Comparison of the safety of changing lane.

The driving safety of vehicles is usually quantitatively compared and analyzed with indicators such as TTC, and in this paper, two-dimensional TTC safety indicators^25^ are used to portray the game decision-making behavior and driving safety of vehicles. As shown in Fig. 12 by statistically analyzing and comparing the lane-changing safety data of ramp vehicles at the time of merging into the mainline lane with the surrounding vehicles, the distribution of the TTC values of the vehicle lane-changing and merging under different models is obtained. The mean value of the TTC in the dataset scenarios is 7.01 s, with a standard deviation of 5.15 s. The mean value of the distribution of the TTC of the SL2015 lane-changing model is 6.39 s, with a standard deviation of 4.86 s. The mean value of TTC of the lane change and cut-out model constructed in this paper is 13.78s, and the standard deviation is 4.74s. Due to the existence of other interactions in the dataset scenarios, the value of TTC in the dataset scenarios is lower than that in the model constructed in this paper, and the latter improves the vehicle collision risk time by an average of 6.77s in comparison to the dataset scenarios and improves it by 7.39s in comparison to the SL2015 lane change model.

The TTCs of the dataset scenarios and SL2015 model are clearly distributed in smaller values. The model TTC values in this paper are large and only a small portion of the data is distributed in smaller values. The simulation results show that the cut-out merging model based on the game perspective portrays the vehicle interaction process in the actual scenario and effectively improves the safety of the vehicle lane changing, the cut-out behavior of the mainline vehicle transforms the game interaction object of the merging vehicle, and increases the vehicle collision time through the staggered alternation of the mainline vehicle to reduce the driving risk of the merging vehicle in the lane changing progress.

### Comparison of vehicle speed on merging section

**Fig. 13:**
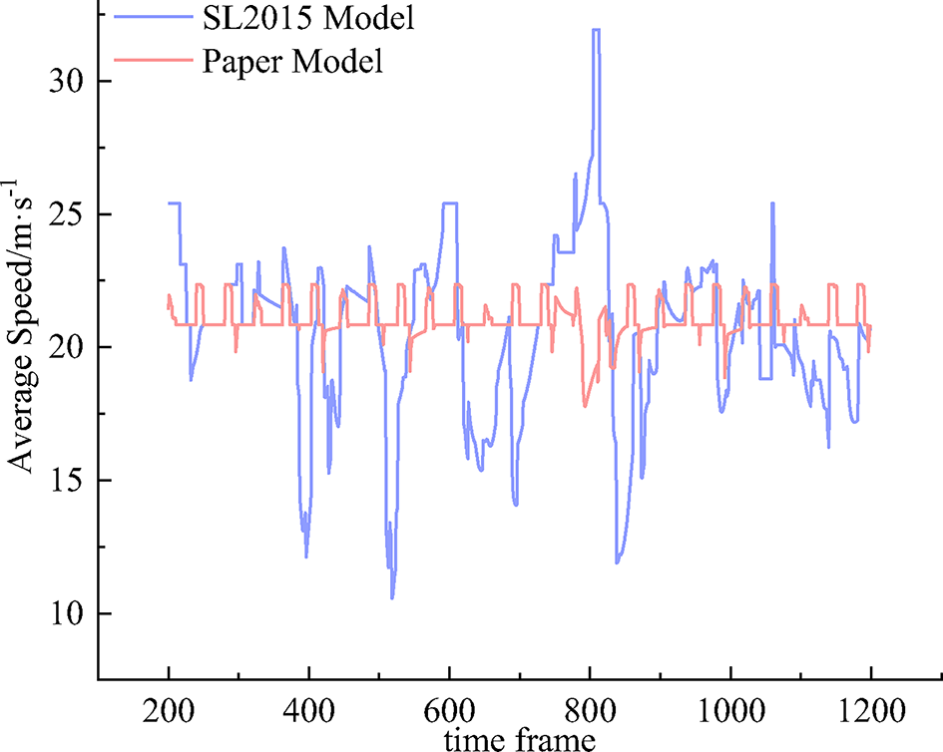
Comparison of average vehicle speed on merging section.

Figure 13 shows the average speed of vehicles in the outer lane of the mainline of the merging section. The simulation results show that the average speed of SL2015 model is 20.49 m/s and the standard deviation is 3.22 m/s, and the average speed of lane change and cut-out and merging model is 21.05 m/s and the standard deviation is 0.75 m/s. The average speed of the former is lower than that of the latter and has higher volatility, indicating that the traffic situation of the merging section in the model changes significantly with time while the vehicle traveling condition of the latter is more stable. Since the starting speed of the vehicle is set during the simulation and the vehicle traveling process is dynamically changing, the average speed in this case varies within a certain range depending on the traveling speed of the vehicles.

Due to the SL2015 model scenario part of the ramp vehicles are forced to travel to the end of the acceleration lane, which results in localized congestion and lane bottles at the end of the merging section, thus significantly reducing the average speed of the mainline vehicles, while the average speed of the vehicles is significantly improved when the congestion is relieved. Based on the trajectory data and vehicle evolution game characteristics of the vehicle lane change and cut-out and merging model of the ramp vehicle, the mainline vehicles can effectively carry out collaborative interaction, to alleviate the vehicle decision-making conflict. At the same time, it can reduce the mutual influence of vehicles, prompting the ramp vehicles to smoothly merge into the mainline lane.

### Vehicle gaming interaction process

**Fig. 14:**
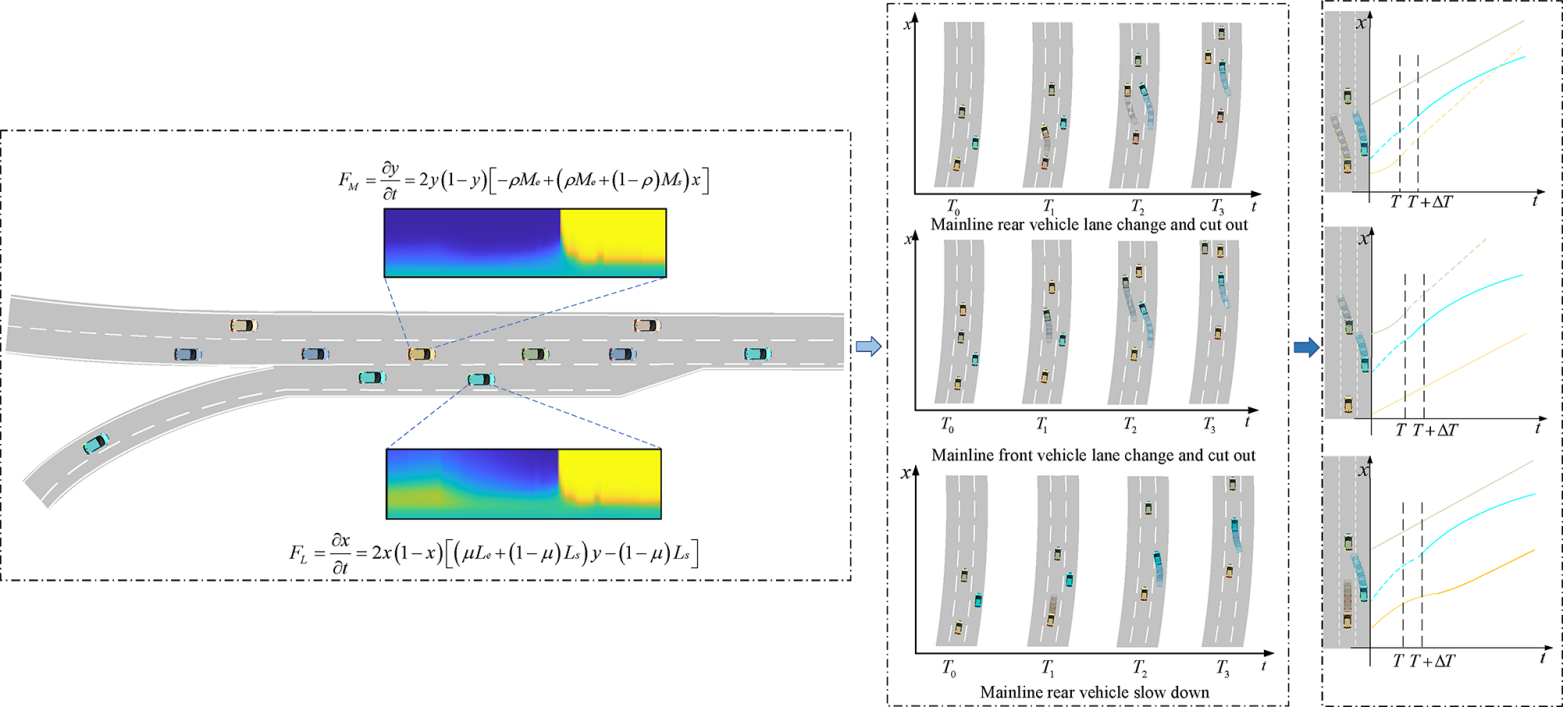
The process of vehicle decision-making behavior.

Based on the evolutionary game theory, it can be known that the vehicle’s game stabilization strategy combinations will converge in different directions at different locations. By combining the vehicle lane change and cut-out and merging behavioral features in the trajectory data, it can be further proved that the game change and interaction process of the vehicle can be further demonstrated. The results of vehicle interactions considering vehicle merging locations, and the mainline vehicle decision cost factors are summarized in Fig. 14. The left part of the figure shows the optimal decision combinations of mainline and ramp vehicles at different merging locations, i.e., ramp vehicles merging and mainline vehicles yielding, ramp vehicles not merging and mainline vehicles not yielding. The middle and right sides of the figure show the driving process of the vehicle choosing different decisions. After making a yield decision, the mainline vehicle chooses whether to change lane and cut out or slow down based on the costly cost of the decision.

According to the dynamic evolution equation of the vehicle game and the convergence result of decision making, the yellow area represents the optimal strategy combination of ramp vehicle changing lane to merge in and mainline vehicle yielding to the vehicle, and the blue area represents the optimal strategy combination of ramp vehicle not merging in and mainline vehicle not yielding to the vehicle. When the mainline vehicle is vulnerable during the evolutionary game, choosing to slow down and yield is the optimal strategy to cope with the situation, but the mainline vehicle’s choice of slowing down to ensure safety gains significantly increases the efficiency cost. For mainline vehicles, choosing lane change and cut-out when the inside lane satisfies the lane change condition is superior to the deceleration strategy, hence the phenomenon of mainline vehicle lane change and cut-out in the trajectory data. Thus, it explains that some of the mainline vehicles avoid the loss of driving efficiency gains caused by being in a weak position during the evolutionary game and choosing to give way by changing lane and cutting-out.

Although the results of the evolutionary game show that the vehicles’ decisions change dynamically, if ramp vehicles cannot complete the merging process at the start position of the ramp, they will be in a disadvantageous position for a significant distance in the acceleration lane, so the ramp vehicles expects tomerge into the mainline lane as soon as possible. And the mainline vehicles know each other’s traveling expectation, therefore, to avoid conflict with them, they should change lane and cut out when the inside lane meets the condition. The choice of lane change and cut-out by mainline vehicles in certain situations is superior to deceleration and yielding to others, and is more favorable to the subsequent game interaction with mainline vehicles.

## Conclusion

By analyzing the driving data of merging vehicles in the exiD high precision trajectory dataset, there exists the phenomenon of sudden change of gap when vehicles change lane to merge, and analyzing the process of lane-changing and cutting out of the mainline vehicles based on the game decision-making and cost perspectives. This study expands the types of divisions of lane-changing and merging of vehicles and the traditional game interaction behavior perspectives of the existing studies.

Vehicle travel speed has a direct impact on the mainline vehicle decision, when the mainline vehicle travel speed is larger, the ramp vehicle’s merging behavior will significantly increase the speed cost of the mainline vehicle. When the mainline vehicle is traveling at a higher speed than the ramp vehicle, the cost of choosing to change lane and cut out is lower than that of slowing down and yielding to the vehicle strategy. The merging position of ramp vehicles is also an important factor affecting the vehicles’ decision to merge in the direction of the ramp, ramp vehicles have different game results for changing lane to merge in different positions of the acceleration lane, and the evolutionary game theory suggests that the game advantages of the ramp vehicles and the mainline vehicles are not fixed, but change with the positional changes. Using the vehicle merging position factor as a macro-influencing factor can verify the results of the mainline vehicles’ lane-changing and cutting-out behavior to avoid themselves being in a non-dominant situation in the future, and therefore achieve the macro-micro consistency of the vehicles’ decision-making behaviors.

The proposed model can not only reveal the special effects of vehicle interaction in the merging section of the highway but also significantly reduce the risk of vehicle collision, and reduce the time of vehicle lane changing and merging, which can provide a reference for related research. In the evolution of the vehicle game strategy, ramp vehicles can always find the optimal gap to change lane, and the different coping strategies of the mainline vehicles show their tendency to judge the efficiency and safety trade-offs. This paper quantitatively analyzes and reproduces the interaction process of highway merging section vehicle game by constructing a game model, instead of adopting a scheduling method based on upper-level macro-control, which can more objectively reflect the interaction process of the game of vehicles in the real scenario.

## Discussion

By combining high-precision trajectory datasets and visualization tools, we mined and analyzed the hidden vehicle interaction features and driver decision-making tendencies in the datasets, and used them as a basis to provide the basis and reference for modeling the interaction behaviors of autonomous Vehicles, and make it fully consider the vehicle’s respective driving needs and macro-collaborative functions.

Quantitatively analyze ramp vehicles’ trade-offs between safety and efficiency in pre-selecting optimal merge gap and meeting vehicle travel expectations before change lane to merge through evolutionary game theory. Mainline vehicles respond to this behavior of ramp vehicles according to their driving speed and safety, and select the best lane to drive in by judging the safety of lane changing and the cost of vehicle driving efficiency in the inside lane. Since the mainline and ramp traffic are continuous, in the evolutionary game analysis, the mainline vehicles are trying to avoid the future loss of efficiency gains due to being in a non-dominant situation. Therefore, in the initial stage of the merging section, the mainline vehicles can only choose the give way strategy. In the yielding outcome, the mainline vehicles can choose to yield by slowing down or cutting out by lane changing. In some cases, the choice of lane changing and cutting out by mainline vehicles is preferred to slowing down, which is more conducive to subsequent game interactions with mainline vehicles. By inverse reasoning it is possible to verify the rationality of the mainline vehicle to change lane and cut out, therefore, models based on micro decision costs and non-cooperative evolutionary games that take macro factors into account are informative for verifying the consistency of vehicle decisions at both macro and micro levels.

By reviewing the research work in this paper, we have made some assumptions and overlooked some corner issues in the modeling process. In future research, we will further explore and investigate the following issues. The vehicle trajectory dataset not only records the traveling state data of the vehicle, but also provides the map data of the road section where the dataset is taken. Through the map visualization tool, it can be found that the dataset road used in this paper has a certain curvature, and the curvature of the road section has a certain effect on the convergence behavior of ramp vehicles. In the subsequent research, it is necessary to further explore the influence of road curvature on the phenomenon of vehicle interaction behavior found in this paper on this basis.

The trajectory dataset is a valuable treasure for us to study the vehicle interaction behavior, which contains the decision tendency of experienced drivers in different situations and even in the corner case. This paper only analyzes and explores part of the interaction behavior, and there are still unexplored features of vehicle interaction behavior, and the subsequent research can still use this dataset as the data base to provide an effective reference for Connected Autonomous Vehicle interaction behaviors modeling.

## Data Availability

The data and vehicle driving images that support the findings of this study are openly available in [The exiD dataset: A real-world trajectory dataset of highly interactive highway scenarios in germany.] at https://doi.org/10.1109/IV51971.2022.9827305, reference number^19^.
